# Colon microbiota and metabolite potential impact on tail fat deposition of Altay sheep

**DOI:** 10.1128/spectrum.03103-23

**Published:** 2024-04-22

**Authors:** Meng Hou, Mengjun Ye, Xuelian Ma, Yawei Sun, Gang Yao, Liya Liu, Xin Li, Yan Hu, Jinquan Wang

**Affiliations:** 1 College of Veterinary Medicine, Xinjiang Agricultural University, Urumqi, China; 2 Institute for Quality & Safety and Standards of Agricultural Products Research, Jiangxi Academy of Agricultural Sciences, Nanchang, China; 3 Institute of Veterinary Medicine, Xinjiang Academy of Animal Science Animal Medical Research Center, Urumqi, China; 4 Animal Disease Control and Diagnosis Center of Altay Region, Xinjiang, China; 5 Technology Talent Development Center of The Xinjiang Uygur Autonomous Region, Urumqi, China; University of Arkansas, Fayetteville, Arkansas, USA

**Keywords:** Altay sheep, tail fat deposition, colon, microbiota, metabolite

## Abstract

Tail fat deposition of Altay sheep not only increased the cost of feeding but also reduced the economic value of meat. Currently, because artificial tail removal and gene modification methods cannot solve this problem, it is maybe to consider reducing tail fat deposition from the path of intestinal microbiota and metabolite. We measured body weight and tail fat weight, collected the serum for hormone detection by enzyme-linked immunosorbent assay, and collected colon contents to 16S rRNA sequence and liquid chromotography with mass spectrometry detection to obtain colon microbiota and metabolite information, from 12 3-month-old and 6-month-old Altay sheep. Subsequently, we analyzed the correlation between colon microbiota and tail fat weight, hormones, and metabolites, respectively. We identified that the tail fat deposition of Altay sheep increased significantly with the increase of age and body weight, and the main microbiota that changed were Verrucomicrobia, Cyanobacteria, *Akkermansia*, *Bacteroides*, *Phocaeicola*, *Escherichia-Shigella*, and *Clostridium_sensu_stricto_1*. The results indicated that the diversities of metabolites in the colon contents of 3-months old and 6-months old were mainly reflected in phosphocholine (PC) and phosphatidylethanolamine (PE) in the lipid metabolism pathway. The correlations analyzed showed that Verrucomicrobia, Chlamydiae, *Akkermansia*, *Ruminococcaceae_UCG-005*, *Bacteroides*, and *Phocaeicola* were negatively correlated with tail fat deposition. Verrucomicrobia, *Akkermansia*, and *Bacteroides* were negatively correlated with growth hormone (GH). Verrucomicrobia was positively correlated with L-a-lysophosphatidylserine and PE(18:1(9Z)/0:0). Our results showed that tail fat deposition of Altay sheep was probably correlated with the abundance of Verrucomicrobia, *Akkermansia*, *Bacteroides* of colon microbiota, PC, PE of metabolites, and GH of serum.

**IMPORTANCE:**

Excessive tail fat deposition of Altay sheep caused great economic losses, and the current research results could not solve this problem well. Now, our research speculates that the tail fat deposition of Aletay sheep may be related to the abundance of Verrucomicrobia, Akkermansia, Bacteroides, metabolites phosphocholine, phosphatidylethanolamine, and growth hormone of serum. Further investigation of the interaction mechanism between these microbiota or metabolites and tail fat deposition is helpful in reducing tail fat deposition of Altay sheep and increasing the economic benefits of breeding farms.

## INTRODUCTION

As a geographical indication product of Xinjiang, Altay sheep is famous for its large size and high meat fat production performance. Adult female Altay sheep weigh 75 kg, and males weigh 100 kg. Compared to the weight of other breeds of adult sheep, the Altay sheep have a significant advantage in weight, such as the adult female Hu sheep weigh 53 kg, and males weigh 85 kg (1). However, Altay sheep are fat-tailed sheep (2–4), and studies have shown that fat deposition requires more energy intake than lean tissue growth, increasing feeding costs (5); the tail fat weight accounts for 20% of the carcass weight (6), which significantly reduces the economic value of meat (7). Therefore, reducing tail fat deposition is an important goal for the Altay sheep to improve profitability (8).

Due to the adverse effects of tail fat deposition, researchers conducted an in-depth exploration of fat-tailed sheep breeds and found that their tail fat deposition rate was significantly higher than that of other parts of the body (9). This led to a large number of studies on the removal of tail fat. Surgical resection or rubber bands were used at the beginning (10–12). Since this method required a large amount of human resources, modification from the perspective of genes was considered feasible. Studies have shown that the expression of fat mass and obesity-associated (FTO) gene in the small-tail group was significantly higher than that in a large-tail group of Hu sheep, and new polymorphic sites of the FTO gene can be used as potential molecular markers for breeding small-tailed sheep (13). High mobility group AT hook 1 (HMGA1) can be used as a candidate gene for Hu sheep breeding and fat-tailed sheep improvement (14). Previous studies in our laboratory also showed that the decrease of FTO gene expression and the increase of leptin gene expression were significantly correlated with the deposition of tail fat in Altay sheep. However, the specific mechanism of gene regulation of tail-fat deposition needs to be further studied, and there is still a certain distance from practical application. Tail fat deposition is not only related to genes, but also some scholars believe that intestinal microbiota play a corresponding role.

Gut microbiota are essential to the normal development and function of many aspects of animal biology (15). Moreover, the gut microbiota have shown that they are associated with fat deposition in some animals, such as chicken (16), pigs (17), and goats (11). The previous studies have shown that the rumen and ileum serve as indispensable fermentation sites in goats (11). Meanwhile, studies have found that there is also a fermentation process in the colon that increases the production of fatty acids (18). Some studies found that *Lachnospiraceae* and *Akkermansia* of small intestine, ileum, and cecum microbiota might distinguish between small-tailed Han sheep and large-tailed Han sheep, and their metabolic process may be involved in the tail fat formation of sheep (15). In addition, the previous studies of our laboratory found that the abundance of *Akkermansia* and *unclassified-f-Lachnospiraceae* in the colon of Altay sheep was higher than that in the small intestine (19). At present, there are no reported studies on the correlation between tail fat deposition and intestinal microbiota of other breeds, and there are no studies on the relationship between colon microbiota and tail fat deposition. Based on the above background studies, we speculated that there may be a correlation between colon microbiota and its metabolites and tail fat deposition in Altay sheep, and there may be key microbiota or metabolites involved in the regulation of tail fat deposition.

In this study, the body weight and tail fat weight of 3-month-old and 6-month-old Altay sheep were measured, respectively, to ensure differences in tail fat deposition. Then, the hormones in serum, microbiota, and metabolites in colon were compared and analyzed, in order to select microbiota or metabolites associated with tail fat deposition. This study provides an alternative scheme for reducing Altay sheep tail fat deposition.

## MATERIALS AND METHODS

### Animal

In the present study, Altay sheep were obtained from a certain Altay sheep breeding farm of Altay. The lambs are breastfed from birth to 45 days of age, supplemented with feed after 45 days of age, weaned at 75 days of age, fed and drank freely from 75 to 90 days of age, and entered the fattening stage after 90 days of age, and the feed ratio was referred to the nutritional requirements of meat sheep (NY/T 816-2004, Table 1). We selected 300 Altay sheep with similar birthday age as the experimental group. When they grew to 3 months old, six Altay sheep were randomly selected as the 3 months group (14.43 ± 2.60 kg), and when they grew to 6 months old, six Altay sheep were randomly selected as the 6 months group (30.50 ± 3.10 kg). In the feeding process, all standardized immunization procedures were used.

**TABLE 1:** The composition and nutrient levels of Altay sheep feed at different ages (%)

	45–90 days	90–180 days
Ingredients		
Alfalfa hay	25.40	–^*c*^
Corn straw	19.40	56.25
Wheat straw	5.00	–
Corn	30.00	29.53
Soybean meal	10.00	3.32
Wheat bran	5.00	6.56
Rapeseed meal	4.00	–
Cottonseed meal	–	2.62
Limestone		0.66
Calcium hydrogen phosphate	0.20	0.18
Calcium carbonate	0.35	–
NaCI	0.50	0.44
Premixture^*a*^	0.15	0.44
Total	100	100
Nutrient levels		
DE (digestible energy)^*b*^	10.66	5.61
CP (crude protein)	12.51	8.14
Ca	0.65	0.48
P	0.45	0.27

^*a*^ The premix of 45–90 days provided the following per kilogram of the diet: Mn: 960 mg, Fe: 800 mg, Zn: 1,600 mg, Cu: 160 mg, Co: 4.00 mg, I: 4.00 mg, VA: 90,000 IU, VD3: 30,000 IU, VE: 300 IU. The premix of 90–180 days provided the following per kilogram of the diet: Cu: 200 mg, Fe: 1,200 mg, Mn: 200 mg, Zn: 400 mg, I: 20 mg, Se: 15 mg, VA: 80,000 IU, VD3: 20,000 IU, VE: 500 IU.

^*b*^ DE (digestible energy) was calculated according to the formula of feed composition.

^*c*^ "-" indicates that the diet does not contain this substance.

### Sample collection

Samples were collected from Altay sheep at 90 and 180 days of age. Before slaughter, the sheep were prevented from consuming feed for 24 h and from drinking for 2 h. First, the body weight of Altay sheep was measured. Second, blood was obtained by the jugular vein method, and collected in additive-free tubes, then centrifuged (3,000 rpm, 15 min) and the obtained serums were stored at −20°C. Then, tail fat was collected and weighed. Finally, colon contents were collected using sterile tubes and stored at −80°C for 16S rRNA sequence and liquid chromotography with mass spectrometry (LC-MS) detection to obtain colon microbiota and metabolite information (19).

### Biochemical analysis

The serum hormone was detected using AU680 automatic biochemical analyzer (Beckman Coulter, Inc., USA). Parameters measured included adiponectin (ADPN), fatty acid-binding protein (FABP4), growth hormone (GH), leptin (LEP), following the instructions strictly. The description or function of hormones is shown in Table 2.

**TABLE 2:** Description of hormones associated with fat deposition

Hormone name	Description or function
Adiponectin	A protein hormone secreted exclusively by adipocytes. Promote the synthesis and activation of adiponectin in fat, and negatively regulate fat deposition (20).
Fatty acid-binding protein 4	A protein expressed in adipose tissue, where it regulates fatty acids storage and lipolysis (21, 22).
Growth hormone	Decreased growth hormone secretion leads to obesity (23).
Leptin	Leptin is primarily secreted by white adipose tissues, and positively regulate fat deposition (24).

### DNA extraction and PCR amplification

Microbial DNA was extracted from the colonic content of Altay sheep samples using the Tiangen-Magnetic Soil And Stool DNA Kit (Tiangen, China) according to the manufacturer’s protocols (19). The final DNA concentration and purification were determined by OneDrop OD 1000+ UV-vis spectrophotometer (Wins, China), and DNA quality was checked by 1% agarose gel electrophoresis. The V3-V4 hypervariable regions of the bacteria 16S rRNA gene were amplified with primers 338F (5′-ACTCCTACGGGAGGCAGCAG-3′) and 806R (5′-GGACTACHVGGGTWTCTAAT-3′) (25) by thermocycler PCR system (GeneAmp 9700, ABI, USA). The PCR reactions were conducted using the following program: 3 min of denaturation at 95°C, 27 cycles of 30 s at 95°C, 30 s for annealing at 55°C, and 45 s for elongation at 72°C, and a final extension at 72°C for 10 min. PCR reactions were performed in triplicate 20 µL mixture containing 4 µL of 5× FastPfu buffer, 2 µL of 2.5 mM dNTPs, 0.8 µL of each primer (5 µM), 0.4 µL of Fast Pfu polymerase, and 10 ng of template DNA. The resulting PCR products were extracted from a 2% agarose gel and further purified using the AxyPrep DNA Gel Extraction Kit (Axygen Biosciences, Union City, CA, USA) and quantified using QuantiFluor-ST (Promega, USA) according to the manufacturer’s protocol. Purified amplicons were pooled in equimolar and paired-end sequenced (2 × 300) on an Illumina Miseq platform (Illumina, San Diego, USA) according to the standard protocols by Majorbio Bio-Pharm Technology Co. Ltd. (Shanghai, China).

### LC-MS detection of colon contents

Fifty milligrams from each colonic content sample was precisely pipetted into 400 µL of cold methanol solution (methanol:ddH_2_O = 3:1) and cryogenically ground with a high-throughput tissue grinder. After vortexing, the samples were sonicated on ice, for extraction, for 10 min (repeated three times), incubated at −20°C for 30 min, and then centrifuged at 12,000 rpm and 4°C for 15 min; the supernatant was tested on the machine.

Twenty microliters of each sample was injected into the HPLC tandem time-of-flight mass spectrometry UPLC-TripleTOF system (AB Sciex). Metabolites were separated using a chromatographic column (100 mm × 2.1 mm, 1.7 µm; Waters, Milford, USA). Mobile phase A was water (containing 0.1% formic acid), and mobile phase B was acetonitrile/isopropanol (1/1) (containing 0.1% formic acid). Metabolites were eluted with the following gradient: 0 min, 5% B; 3 min, 20% B; 9 min, 95% B; 13 min, 95% B; 13.1 min, 5% B; and 16 min, 5% B delivered at a rate of 0.4 mL/min; the column temperature was set to 40°C. The MS signal acquisition mode was positive and negative ion scanning, the electrospray capillary voltage was 1.0 kV, the injection voltage was 40 V, and the collision voltage was 6 eV. The ion source temperature and desolvation temperature were 120°C and 500°C, respectively, the carrier gas flow was 900 L/h, the mass spectrometry scanning range was 50–1,000 m/z, and the resolution was 30,000.

### Statistical analysis

The data of body weight, tail fat weight, and serum hormone were analyzed using GraphPad Prism 8 and *t*-tests were used to determine statistical significance. A *P* value <0.05 was considered significant. The sheep’s core microbiota were identified by selecting OTUs that were shared by at least 95% of the samples. The Student’s *t*-test was used to analyze microbial diversity indexes, the Shannon index represents the gut microbiota diversity, and the coverage index represents the coverage of the sample library. Microbial diversity was determined by the Wilcoxon rank-sum test (the confidence interval was 95%). Significant differences were considered at *P* < 0.05 (19). All metabolites identified by mass spectrometry were compared with Kyoto Encyclopedia of Genes and Genomes (KEGG) database to obtain annotation information, and their annotation in the database was statistically analyzed, and orthogonal partial least squares discriminant analysis (OPLS-DA) method was used to analyze the differential metabolites between groups. Correlations among microbiota, metabolites, hormones, and tail fat weight were calculated using Spearman rank correlation coefficients and represented by heatmap.

## RESULTS

### Growth performance and tail fat deposits

**Fig 1:**
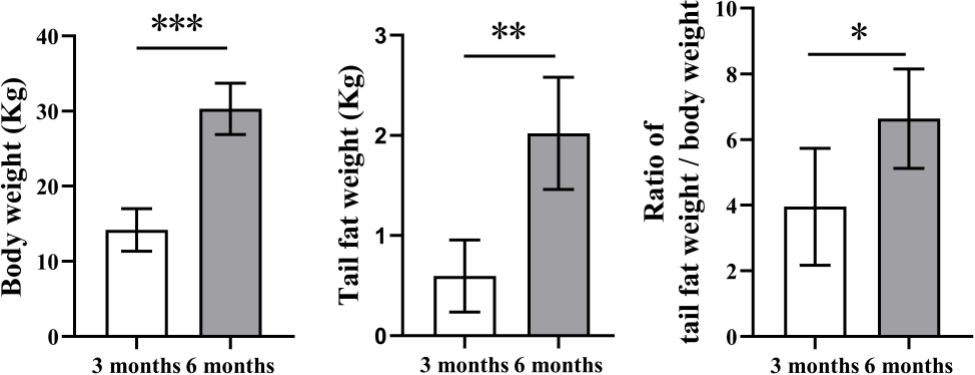
Diversities in body weight and tail fat weight of Altay sheep at different months of age. 3 months, 3-month-old Altay sheep (*n* = 6); 6 months, 6-month-old Altay sheep (*n* = 6). Columns, mean of average value; bars, SD. Statistical significance is indicated by *P < 0.05.

**Fig 2:**
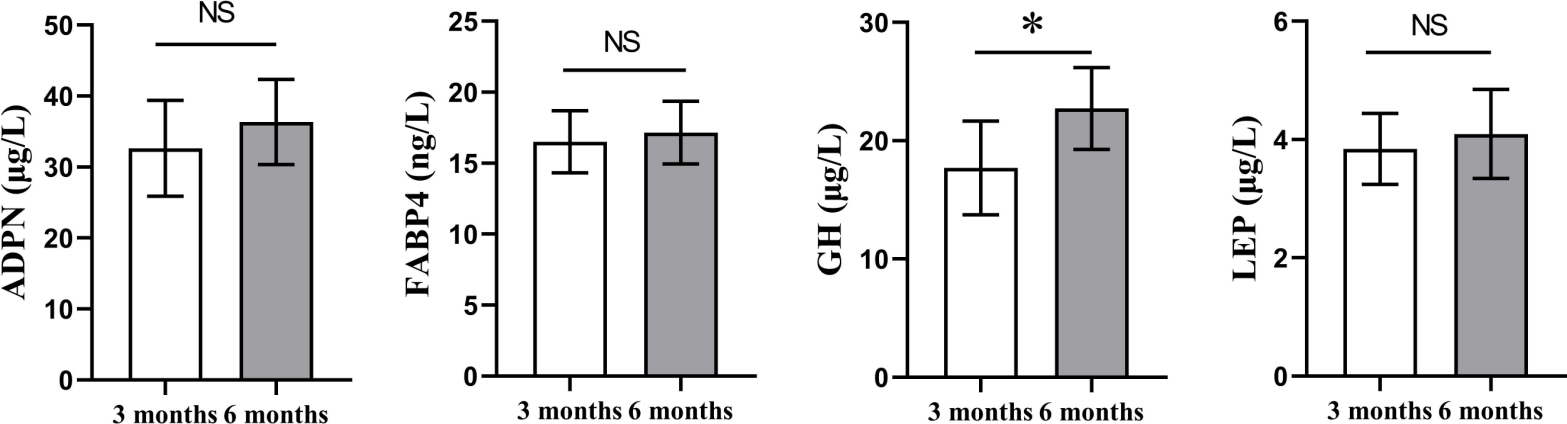
The serum hormone indicators. 3 months, 3-month-old Altay sheep (*n* = 6); 6 months, 6-month-old Altay sheep (*n* = 6). Columns, mean of average value; bars, SD. Statistical significance is indicated by *P < 0.05.

To clarify whether the weight of tail fat increases with the age of Altay sheep, we selected 3-month-old and 6-month-old sheep (*n* = 6), weighed the body weight and tail fat weight, and calculated the ratio of tail fat weight/body weight. We also detected the content of ADPN, FABP4, GH, and LEP in serum by enzyme-linked immunosorbent assay. As shown in Fig. 1, body weight, tail fat weight, and the ratio of tail fat weight/body weight in the 6 months were significantly increased compared with that in the 3 months (*P* < 0.05). GH level was significantly lower in the 3 months than in the 6 months (*P* < 0.05), and no significant changes in other hormone levels (Fig. 2). The above results showed that the ratio of tail fat weight/body weight and the content of growth hormone increased significantly with the increase of body weight of Altay sheep.

### Diversities in the colon microbiota

**Fig 3:**
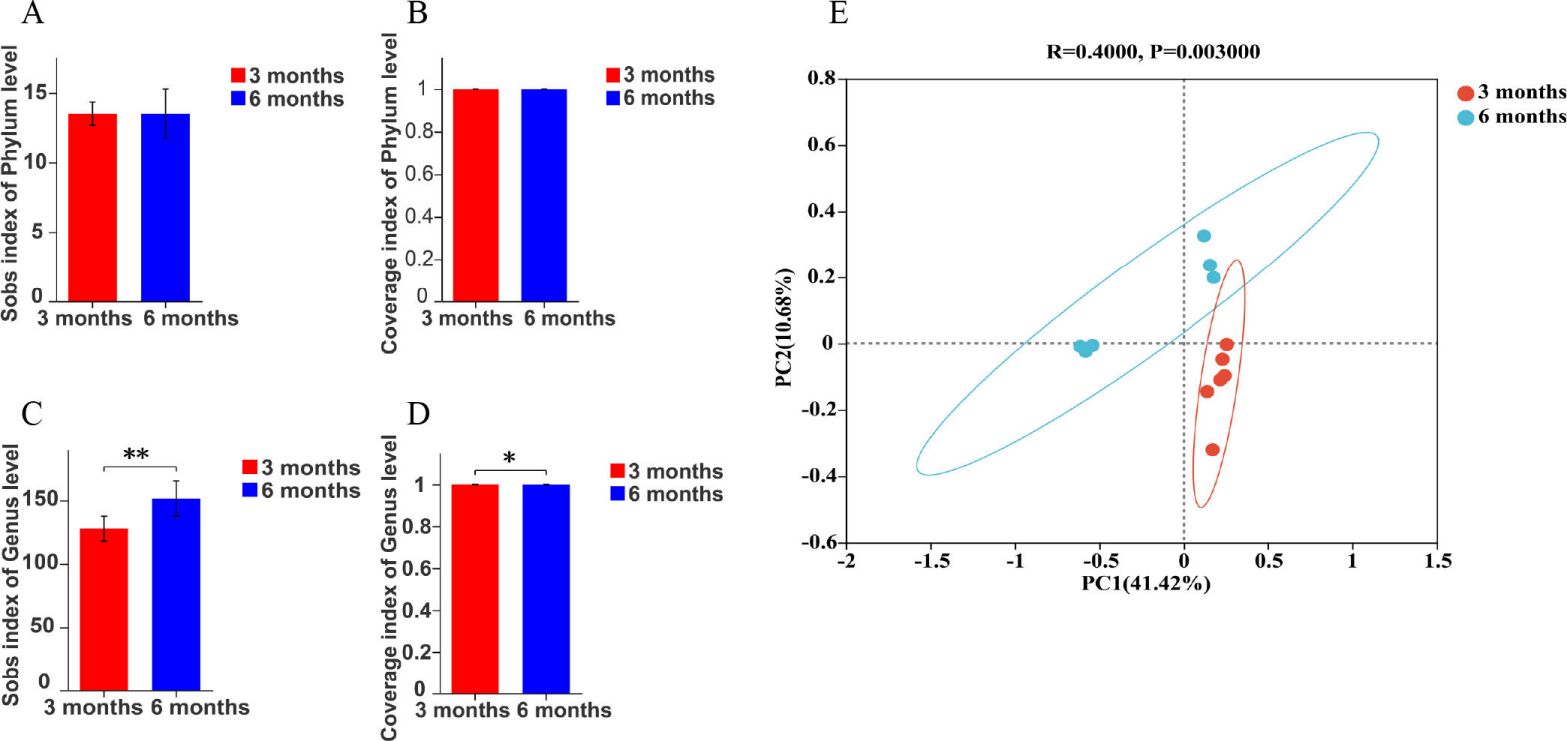
Alpha and beta diversities’ analysis at different months of age. 3 months, 3-month-old Altay sheep (*n* = 6); 6 months, 6-month-old Altay sheep (*n* = 6). (**A**) Sobs index of the phylum level, (**B**) coverage index of the phylum level, (**C**) sobs index of the genus level, (**D**) coverage index of the genus level, and (**E**) PCoA analysis. Columns, mean of average value; bars, SD. Statistical significance is indicated by *P < 0.05, **P < 0.01.

The results showed that the sobs index and coverage index of the two groups were basically consistent at the phylum level (Fig. 3A and B). At the genus level, the sobs index was significantly higher in the 6 months than in the 3 months (Fig. 3C and D). The results of principal co-ordinates analysis (PCoA) analysis in beta diversity showed a significant difference in the composition of colonic microbiota between 3 months and 6 months (Fig. 3E).

**Fig 4:**
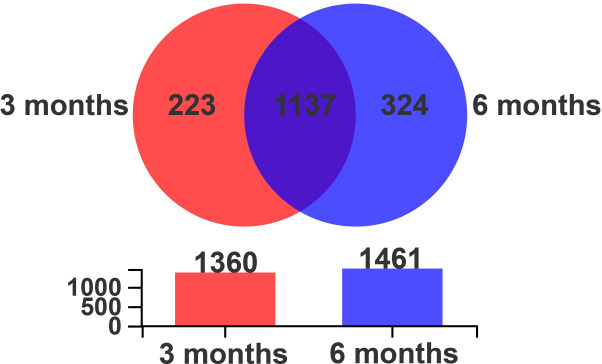
OTUs Venn diagram. 3 months, 3-month-old Altay sheep (*n* = 6); 6 months, 6-month-old Altay sheep (*n* = 6).

**Fig 5:**
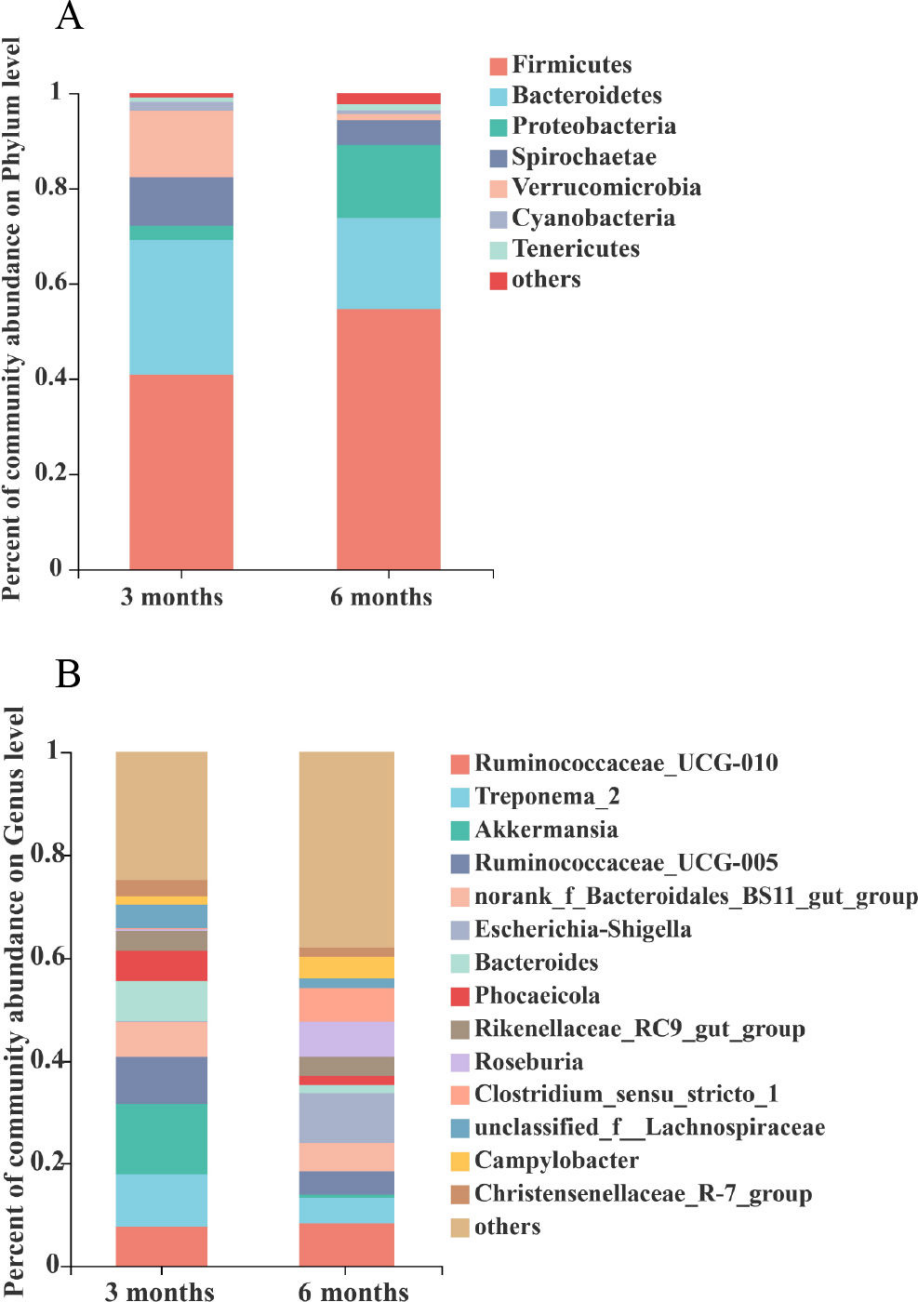
Community abundance of the colon microbiota. (**A**) Phylum level and (**B**) genus level. 3 months, 3-month-old Altay sheep (*n* = 6); 6 months, 6-month-old Altay sheep (*n* = 6).

It can be seen from the Venn diagram that there were 1,360 optical transform unit (OTU) in the 3 months and 1,461 OTU in the 6 months, respectively. The number of unique OTU was higher in the 6 months than in the 3 months (Fig. 4). With the growth of Altay sheep, the species of colon microbiota also changed. The results showed, at the phylum level, higher populations of Bacteroidetes, Spirochaetae, Verrucomicrobia, and Cyanobacteria in the 3 months and higher populations of Firmicutes, Proteobacteria, and Tenericutes were detected in the 6 months (Fig. 5A). Further analysis indicated that at the genus level, *Treponema_2*, *Akkermansia*, *Ruminococcaceae_UCG-005*, *Bacteroides*, *Phocaeicola*, and *unclassified_f_Lachnospiraceae* were higher in the 3 months, and *Ruminococcaceae_UCG-010*, *Escherichia-Shigella*, *Roseburia*, and *Clostridium_sensu_stricto_1* were higher in the 6 months (Fig. 5B).

**Fig 6:**
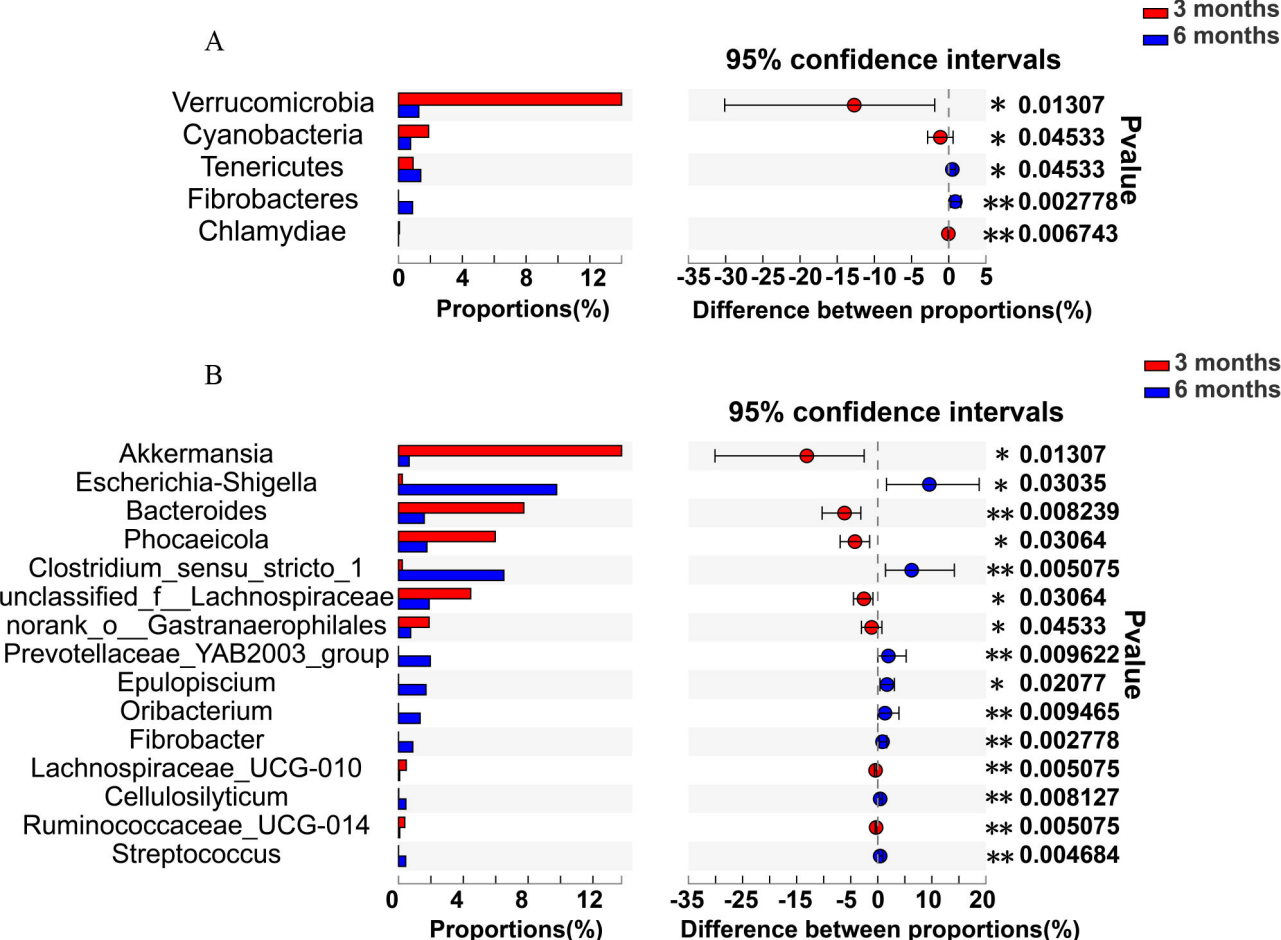
Microbiota diversities of the colon microbiota between the 3 months and 6 months Altay sheep. (**A**) Microbial diversities: phylum level, determined by the Wilcoxon rank-sum test; the confidence interval was 95%. (**B**) Microbial diversities: genus level, determined by the Wilcoxon rank-sum test; the confidence interval was 95%. 3 months, 3-month-old Altay sheep (*n* = 6); 6 months, 6-month-old Altay sheep (*n* = 6). *P < 0.05, **P < 0.01.

As shown in Fig. 6A, at the phylum level, higher populations of Chlamydiae, Verrucomicrobia, and Cyanobacteria were determined in the 3 months than in the 6 months (*P* < 0.05), while higher populations of Fibrobacteres and Tenericutes were detected in the 6 months than in the 3 months (*P* < 0.05). The genus level of *Akkermansia*, *Bacteroides*, *Phocaeicola*, *Unclassified_f_Lachnospiraceae*, *norank_o_Gastranaerophilales*, *Lachnospiraceae_UCG-010*, and *Ruminococcaceae_UCG-014* were higher in the 3 months than in the 6 months (*P* < 0.05), and the *Escherichia-Shigella*, *Clostridium_sensu_stricto_1*, *Prevotellaceae_YAB2003_group*, *Epulopiscium*, *Oribacterium*, and *Fibrobacter* were higher in the 6 months than in the 3 months (*P* < 0.05) (Fig. 6B).

**Fig 7:**
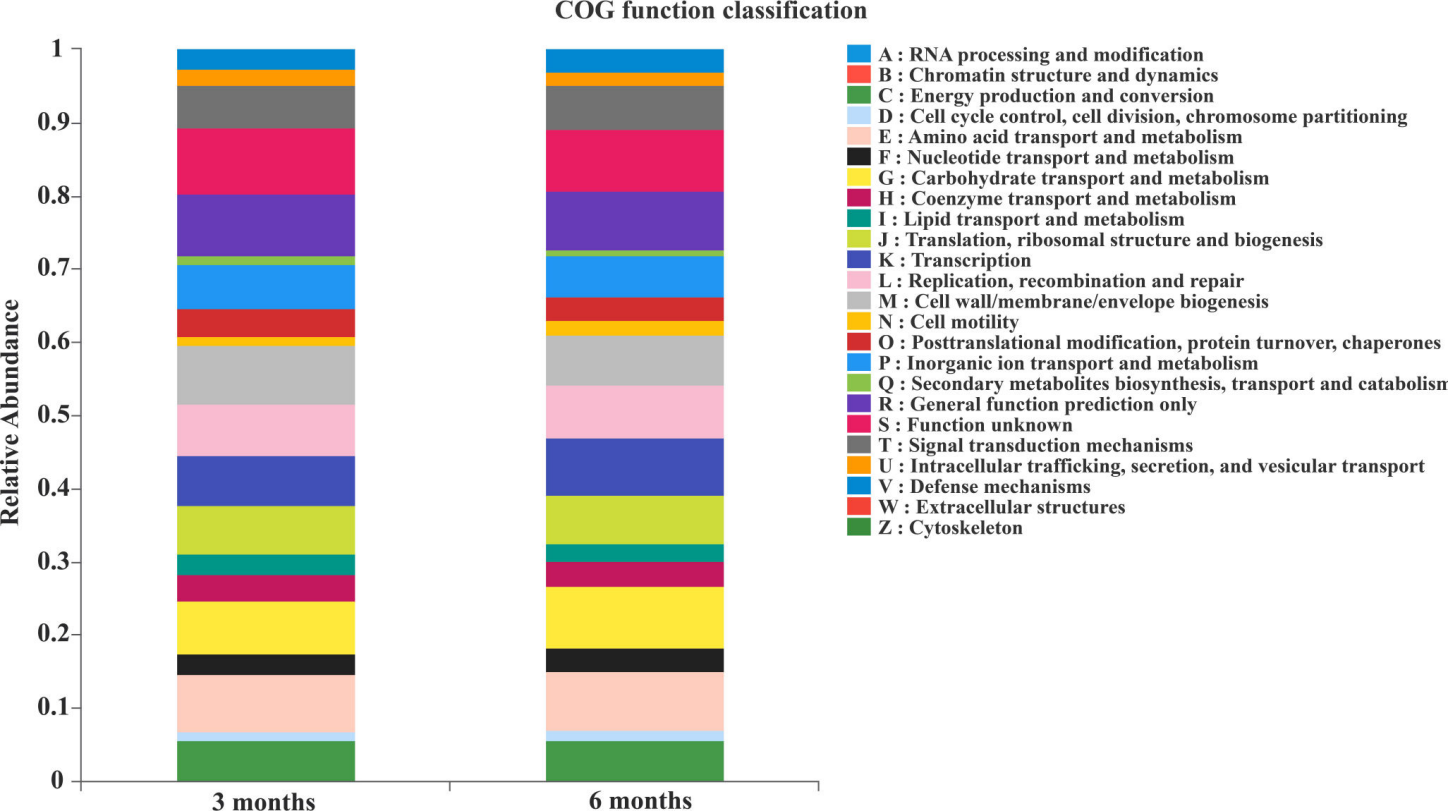
COG function classification of the colonic microbiota. 3 months, 3-month-old Altay sheep (*n* = 6); 6 months, 6-month-old Altay sheep (*n* = 6).

Clusters of orthologous groups (COG) result showed that the functional composition of colon microbiota was similar between the 3 months and the 6 months (Fig. 7). The above results showed that with the growth of Altay sheep, the species of intestinal microbiota would also change correspondingly. The main bacteria that changed were Verrucomicrobia, Cyanobacteria, *Akkermansia*, *Bacteroides*, *Phocaeicola*, *Escherichia-Shigella*, and *Clostridium_sensu_stricto_1*.

### Diversities in the metabolites

**Fig 8:**
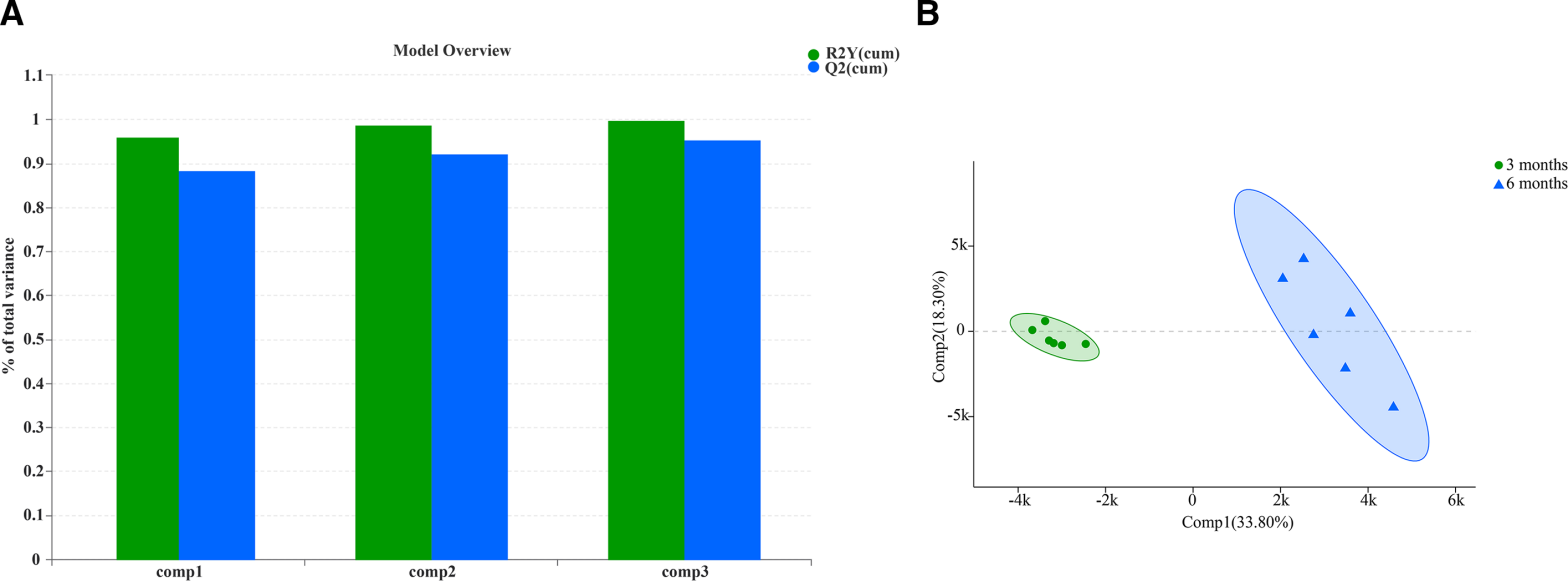
(A) OPLS-DA model overview, (B) OPLS-DA model score.

To determine the diversities of metabolites with age, we collected colon contents of 3 months and 6 months Altay sheep, respectively. The LC-MS method was used to detect and obtain metabolite information. As shown in Fig. 8A, the OPLS-DA model showed that cumulative values of R2Y and Q2Y were close to 1; thus, the constructed model was stable and reliable. The results of PCoA analysis also showed that the various variables screened were the most important and noteworthy metabolites (Fig. 8B).

**Fig 9:**
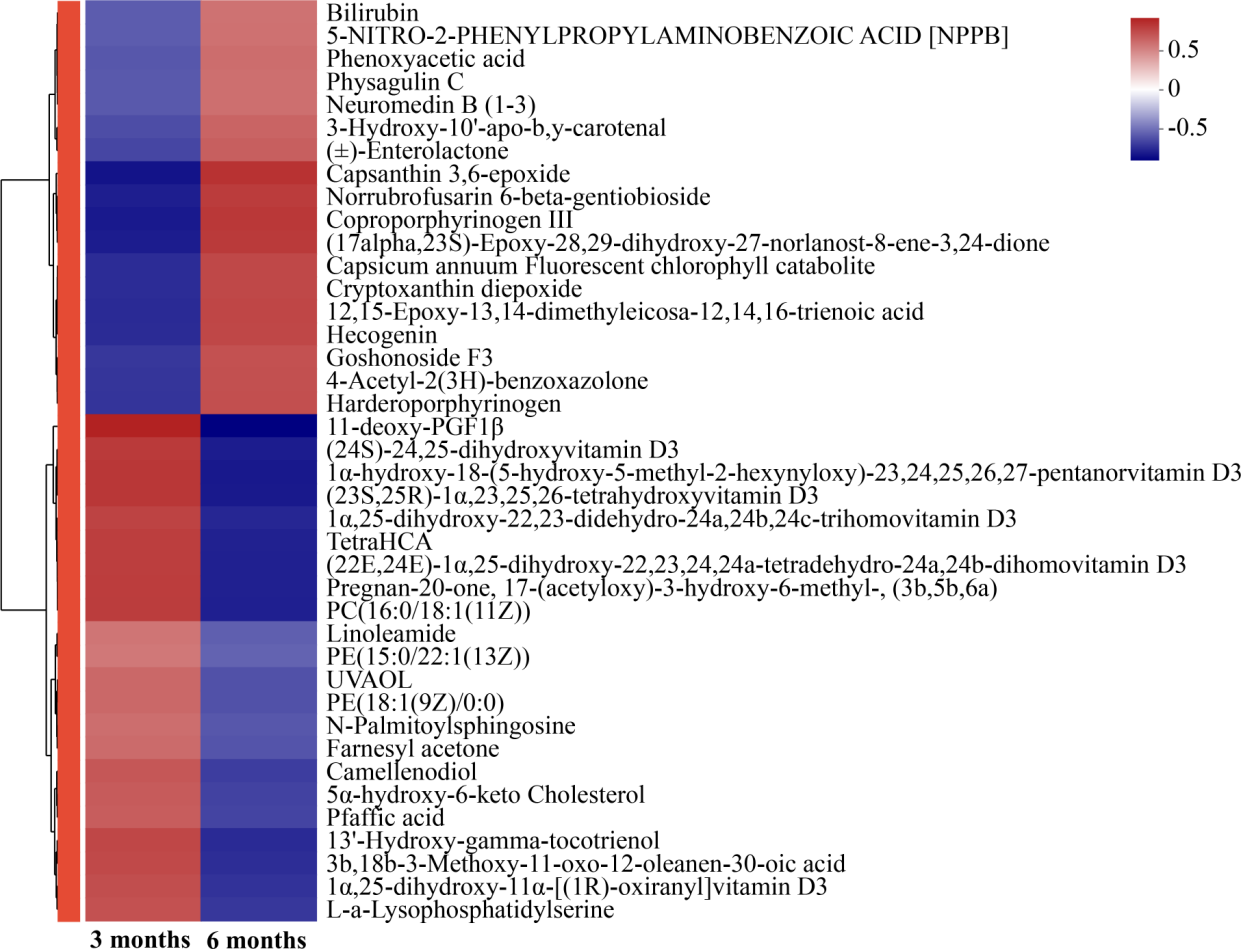
Cluster analysis of various metabolites. 3 months, 3-month-old Altay sheep (*n* = 6); 6 months, 6-month-old Altay sheep (*n* = 6).

A total of 139 metabolites were detected from the colon contents of all experimental animals, and based on the cluster analysis of the top 40 differentially accumulated metabolites, higher levels of 18 metabolites (such as bilirubin) were determined in the 6 months than in the 3 months. Higher levels of 22 metabolites, including phosphocholine (PC): PC(16:0/18:1(11Z)), phosphatidylethanolamine (PE): PE(15:0/22:1(13Z)), and linoleamide, were detected in the 3 months than in the 6 months (Fig. 9).

**Fig 10:**
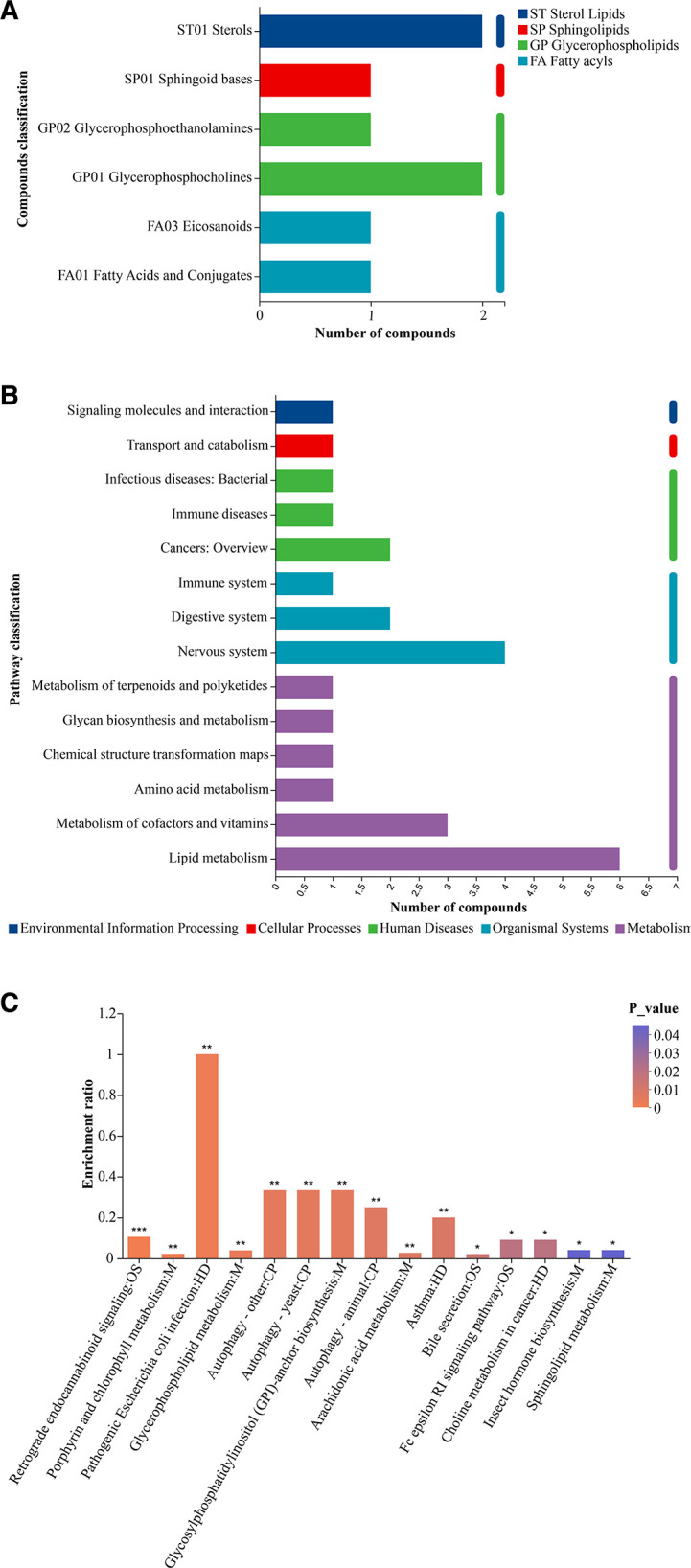
(**A**) Compounds classification of differential metabolites. (**B**) Diverse metabolite KEGG functional pathway. (**C**) KEGG pathway enrichment analysis. The ordinate is the KEGG pathway. *P < 0.05, **P < 0.01.

KEGG compound classification was used to classify the biological functional levels involved in metabolites. We mainly focused on the compounds involved in lipid metabolism. As shown in Fig. 10A, first, we obtained the KEGG Brite primary classification of metabolites, including sterol lipids, sphingolipids, glycerophospholipids, and fatty acyls; then, it showed the secondary classification, including fatty acids and conjugates, eicosanoids, glycerophosphocholines, glycerophosphoethanolamines, sphingoid bases, and sterols; finally, it showed the number of metabolites annotated to the secondary classification and the names of specific metabolites listed in Table 3. The 139 metabolites obtained were put into the KEGG pathway database for comparison, and 26 metabolites were found to participate in five KEGG metabolic pathways, respectively, among which the largest number of metabolites participated in metabolism (Fig. 10B). The specific names of metabolites are shown in Table 4, among which the largest number of metabolites related to lipid metabolism were PC(16:0/20:4(5Z,8Z,11Z,14Z)), PC(16:0/18:1(11Z)), docosapentaenoic acid (22n-3), leukotriene C4, phytosphingosine, and PE(15:0/22:1(13Z)). After the KEGG pathway enrichment analysis, it was found that two metabolites were significantly enriched in the glycerophospholipid metabolic pathway, two metabolites were significantly enriched in the bile secretion pathway, while two metabolites were significantly enriched in the arachidonic acid pathway (*P* < 0.05) (Fig. 10C). The results indicated that the difference of metabolites of 3-months old and 6-months old was mainly reflected in the lipid metabolism pathway, and the main metabolites involved were PC(16:0/20:4(5Z,8Z,11Z,14Z)), PC(16:0/18:1(11Z)), and PE(15:0/22:1(13Z)).

**TABLE 3:** Compound classification statistical

First category^*a*^	Second category^*b*^	Number^*c*^	Metabolite name^*d*^
Fatty acyls	FA01 fatty acids and conjugates	1	Docosapentaenoic acid (22n-3)
Fatty acyls	FA03 eicosanoids	1	Leukotriene C4
Glycerophospholipids	GP01 glycerophosphocholines	2	PC(16:0/20:4(5Z,8Z,11Z,14Z)), PC(16:0/18:1(11Z))
Glycerophospholipids	GP02 glycerophosphoethanolamines	1	PE(15:0/22:1(13Z))
Sphingolipids	SP01 sphingoid bases	1	Phytosphingosine
Sterol Lipids	ST01 sterols	2	Nuatigenin, sarsasapogenin

^*a*^ KEGG Brite primary classification of metabolites.

^*b*^ KEGG Brite secondary classification of metabolites.

^*c*^ The number of metabolites in the corresponding metabolite set annotated to this secondary classification.

^*d*^ The name of the metabolite that is annotated.

**TABLE 4:** KEGG annotation statistics

First category^*a*^	Second category^*b*^	Number^*c*^	Metabolite name^*d*^
Cellular processes	Transport and catabolism	1	PE(15:0/22:1(13Z))
Environmental information processing	Signaling molecules and interaction	1	Leukotriene C4
Human diseases	Cancers: overview	2	PC(16:0/20:4(5Z,8Z,11Z,14Z)), PC(16:0/18:1(11Z))
Human diseases	Immune diseases	1	Leukotriene C4
Human diseases	Infectious diseases: bacterial	1	PE(15:0/22:1(13Z))
Metabolism	Amino acid metabolism	1	PE(15:0/22:1(13Z))
Metabolism	Chemical structure transformation maps	1	Hecogenin
Metabolism	Glycan biosynthesis and metabolism	1	PE(15:0/22:1(13Z))
Metabolism	Lipid metabolism	6	PC(16:0/20:4(5Z,8Z,11Z,14Z)), PC(16:0/18:1(11Z)), docosapentaenoic acid (22 n-3), leukotriene C4, phytosphingosine, PE(15:0/22:1(13Z))
Metabolism	Metabolism of cofactors and vitamins	3	Bilirubin, D-urobilin, coproporphyrinogen III
Metabolism	Metabolism of terpenoids and polyketides	1	Crustecdysone
Organismal systems	Digestive system	2	Bilirubin, leukotriene C4
Organismal systems	Immune system	1	Leukotriene C4
Organismal systems	Nervous system	4	PC(16:0/20:4(5Z,8Z,11Z,14Z)), PC(16:0/18:1(11Z)), leukotriene C4, PE(15:0/22:1(13Z))

^*a*^ First class classification of metabolic pathways.

^*b*^ Secondary classification of metabolic pathways.

^*c*^ The number of metabolites in the corresponding metabolite set annotated to this secondary class.

^*d*^ The name of the metabolite that is annotated.

### Correlation of colon microbiota, metabolites, and tail fat deposition

**Fig 11:**
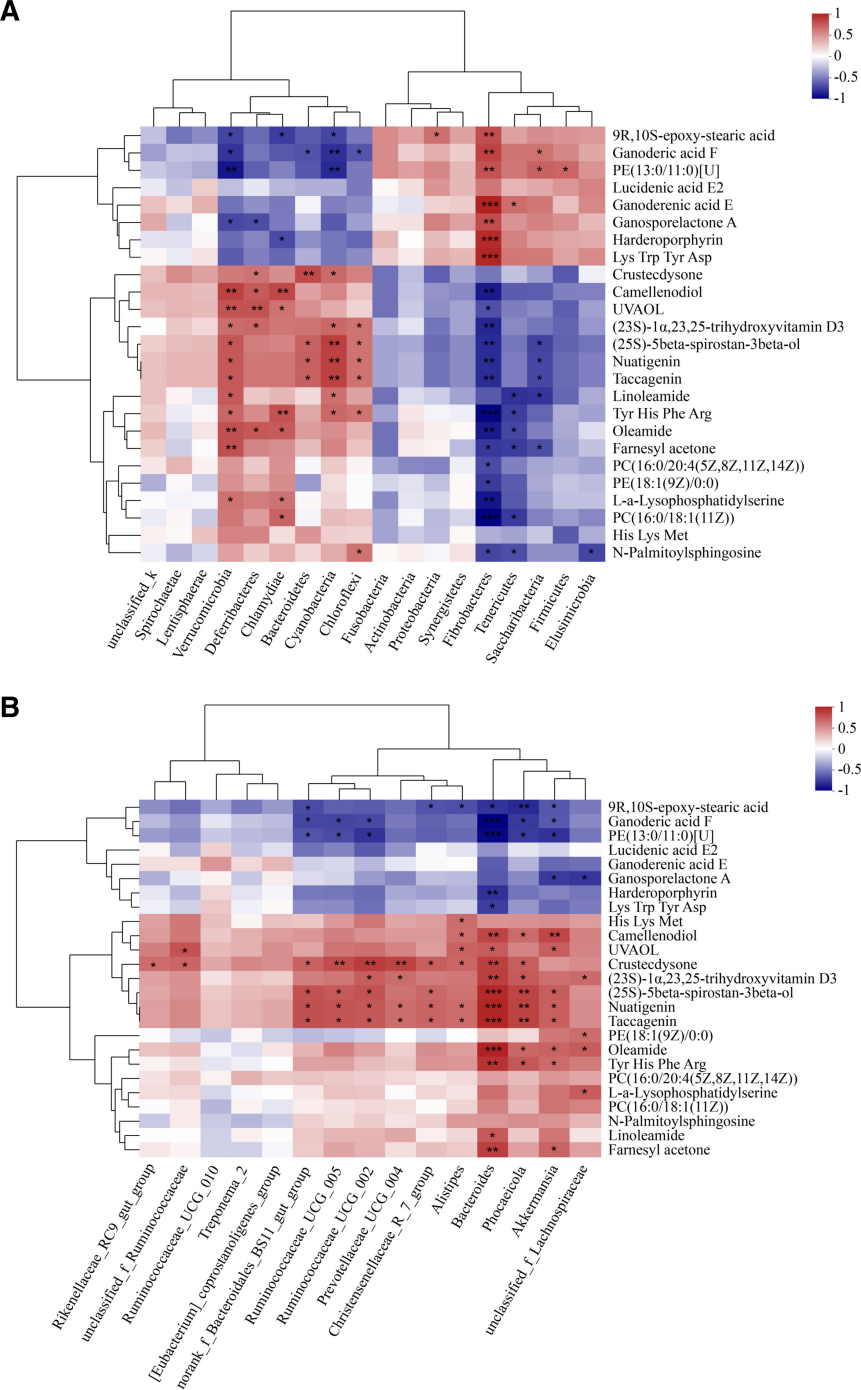
Correlation between colon microbiota and metabolites related. (**A**) Phylum level and (**B**) genus level. Statistical significance is indicated by *P < 0.05, **P < 0.01.

In order to explore the correlation between colon microbiota, metabolites, and tail fat deposition, we sorted out the previous data, calculated the Spearman rank correlation coefficient, and speculated the correlation between them according to *R* and *P* values. We performed an analysis of the correlation between the colon microbiota and metabolites. At the phylum level, Chlamydiae was positively associated with PC(16:0/18:1(11Z)), L-a-lysophosphatidylserine, oleamide, and camellenodiol; Verrucomicrobia was positively associated with L-a-lysophosphatidylserine, (23S)-1α,23,25-trihydroxyvitamin D3, (25S)-5beta-spirostan-3beta-ol and camellenodiol; Cyanobacteria was positively associated with nuatigenin, taccagenin, (23S)-1α,23,25-trihydroxyvitamin D3 and linoleamide; Fibrobacteres was negatively correlated with PC(16:0/18:1(11Z)), L-a-lysophosphatidylserine, PE(18:1(9Z)/0:0), PC(16:0/20:4(5Z,8Z,11Z,14Z)), and camellenodiol; Tenericutes was negatively correlated with PC(16:0/18:1(11Z)) and N-palmitoylsphingosine (Fig. 11A). At the genus level, L-a-lysophosphatidylserine and PE(18:1(9Z)/0:0) were positively associated with *Unclassified_f_Lachnospiraceae*; PE(13:0/11:0)[U] was negatively correlated with *Akkermansia*, *Phocaeicola*, and *Bacteroides*; nuatigenin and taccagenin were positively associated with *Akkermansia*, *Phocaeicola*, *Bacteroides*, and *Ruminococcaceae_UCG-005*; (23S)-1α,23,25-trihydroxyvitamin D3 was positively associated with *Unclassified_f_Lachnospiraceae*, *Phocaeicola*, *Bacteroides*, *Ruminococcaceae_UCG-005*, and *Prevotellaceae_UCG-004* (Fig. 11B).

**Fig 12:**
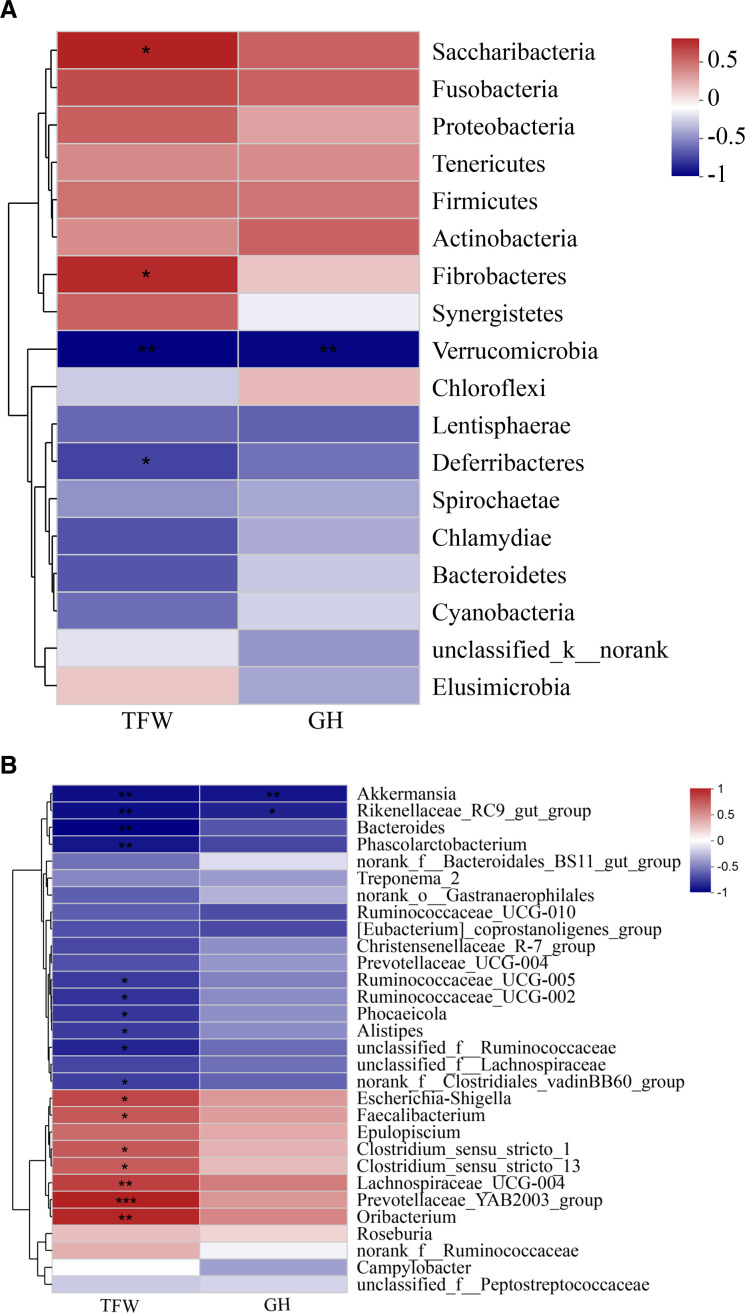
Correlation between tail fat deposition, GH, and colon microbiota related. (**A**) Phylum level and (**B**) genus level. Statistical significance is indicated by * *P* < 0.05, ** *P* < 0.01.

We used Spearman rank coefficient to analyze the correlation between colon microbiota, tail fat weight, and GH content. As shown in Fig. 12A, at the phylum level, Verrucomicrobia was negatively associated with tail fat weight and GH content; Fibrobacteres and Saccharibacteria were positively associated with tail fat weight. At the genus level, *Akkermansia* was negatively associated with tail fat weight and GH content, *Bacteroides*, *Ruminococcaceae_UCG-002*, *Ruminococcaceae_UCG-005*, *Phocaeicola*, and *Unclassified_f_Lachnospiraceae* were negatively associated with tail fat weight; *Clostridium_sensu_stricto_1*, *Faecalibacterium*, *Oribacterium*, *Clostridium_sensu_stricto_13*, and *Lachnospiraceae_UCG-004* were positively correlated with tail fat weight (Fig. 12B).

**Fig 13:**
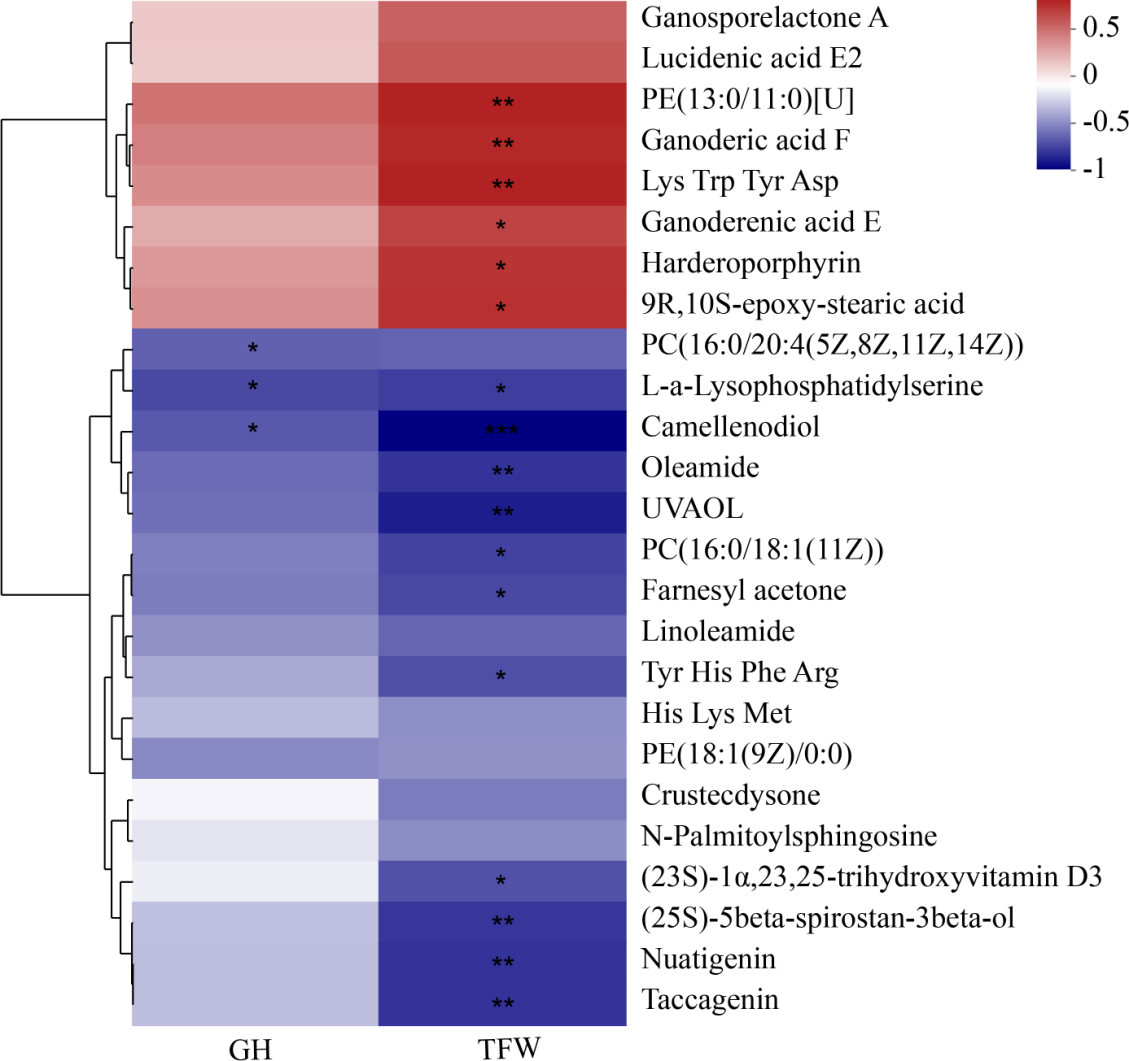
Correlation between tail fat deposition, GH, and metabolites related. Statistical significance is indicated by * *P* < 0.05, ** *P* < 0.01.

Then, we analyzed the correlation between metabolites, tail fat weight, and GH content. The result is shown in Fig. 13. PC(16:0/20:4(5Z,8Z,11Z,14Z)), L-a-lysophosphatidylserine, and camellenodiol were negatively associated with GH content; PE(13:0/11:0)[U] and 9R,10S-epoxy-stearic acid were positively correlated with tail fat weight; L-a-lysophosphatidylserine, camellenodiol, (23S)-1α,23,25-trihydroxyvitamin D3, PC(16:0/18:1(11Z)), (25S)-5beta-spirostan-3beta-ol, nuatigenin, and taccagenin were negatively associated with tail fat weight.

Based on the above results, we believed that there was a certain correlation between colon microbiota, metabolites, tail fat weight, and GH content. The results indicated that the abundance of Verrucomicrobia, *Akkermansia*, *Bacteroides*, and *Phocaeicola* was positively correlated with PC(16:0/20:4(5Z,8Z,11Z,14Z)), PC(16:0/18:1(11Z)), and PE(18:1(9Z)/0:0); tail fat weight and GH content were negatively associated with the abundance of Verrucomicrobia, *Akkermansia*, *Bacteroides*, and *Phocaeicola*, and they were also negatively associated with PC(16:0/20:4(5Z,8Z,11Z,14Z)), PC(16:0/18:1(11Z)), and PE(18:/(9Z)/(0:0).

## DISCUSSION

In this communication, we found for the first time that the abundance of Verrucomicrobia, *Akkermansia*, and *Bacteroides* in the colon was negatively correlated with the deposition of Altay sheep tail fat. Verrucomicrobia may coordinate with lipid metabolites PC(16:0/20:4(5Z,8Z,11Z,14Z), PC(16:0/18:1(11Z)), and PE(15:0/22:1(13Z)) to regulate tail fat deposition. Our colon microbiota and metabolism results showed that the microbiota abundance of Verrucomicrobia, *Akkermansia*, *Bacteroides*, *Phocaeicola*, and *Ruminococcaceae_UCG-005* in the group with increased tail fat deposition was significantly reduced. At the same time, lipid metabolites PC(16:0/20:4(5Z,8Z,11Z,14Z), PC(16:0/18:1(11Z)), and PE(15:0/22:1(13Z)) were also decreased. The results of the correlation study showed that the microbiota abundance of Verrucomicrobia, *Akkermansia*, *Bacteroides*, *Phocaeicola*, and *Ruminococcaceae_UCG-005* had a significant negative correlation with the weight of tail fat. The abundance of *Akkermansia* and *Bacteroides* was negatively correlated with the contents of GH in serum. The abundance of Verrucomicrobia was positively correlated with the contents of lipid metabolites PC(16:0/20:4(5Z,8Z,11Z,14Z), PC(16:0/18:1(11Z)), and PE(15:0/22:1(13Z)).

Our study showed that the microbiota abundance of Verrucomicrobia, *Akkermansia*, and *Bacteroides* in the colon decreased significantly with age and increased tail fat deposition. However, in the study of intestinal microbiome of Tibetan sheep, it was found that Verrucomicrobia accounted for a relatively small proportion, and its abundance increased significantly with the increase of age (26), contrary to the results of our study. The abundance of *Akkermansia* was significantly reduced in mice fed with high-fat diet (HFD) (27), and a comparative test between mice fed with other HFD and normal feeding showed that the progression of fat deposition was negatively correlated with the abundance of *Akkermansia* (28, 29). In addition, some studies have used *Akkermansia* to treat fat increase and adipose tissue inflammation caused by HFD, and achieved good therapeutic effects (29). Moreover, studies have shown that *Akkermansia* is the only genus of Verrucomicrobia found in gastrointestinal samples, colonizing the mucous layer of the colon and participating in the maintenance of intestinal integrity (30). It has been suggested that there may be a correlation between host fat deposition and gut microbiota, as many bacteria can collect energy from the diet and produce peptides to regulate the absorption of fatty acids (31). Studies have shown that using Bacteroides as probiotics to gavage mice can promote the decomposition of brown fat and reduce the weight of mice (32). In conclusion, Verrucomicrobia, *Akkermansia*, and *Bacteroides* have a negative regulatory effect on tail fat deposition.

Our study showed that GH content was positively correlated with fat deposition and negatively correlated with the abundance of Verrucomicrobia and *Akkermansia*. Researchers have found that GH is related to fat deposition and growth, and when the activity of growth hormone-insulin-like growth factor-1 axis is increased, GH content decreases, thus inhibiting the accumulation of fat deposition (33). Other studies have shown that the abundance of *Akkermansia* is negatively correlated with blood markers such as lipid synthesis and obesity (27, 34). It can be found that GH, tail lipid deposition and Verrucomicrobia, *Akkermansia* are correlated, but the specific mechanism of action still needs to be further explored.

The metabolic results showed that PC and PE contents were negatively correlated with tail fat deposition. In the mice experiment with HFD intervention, PC content was reduced (35). Other studies have also shown that the levels of PC and PE in mice fed HFD are reduced (36). Moreover, the correlation results confirmed that the abundance of Verrucomicrobia was positively correlated with the contents of PC and PE. The previous results also showed that Verrucomicrobia had a negative regulatory effect on tail lipid deposition, so Verrucomicrobia and the lipid metabolites PC and PE may work together to reduce tail fat deposition. However, the relationship between gut microbiota and metabolites needs to be further explored to provide new insights into how to help reduce tail fat deposition.

### Conclusion

The results showed that the fat deposition of Altay sheep may be related to the abundance of Verrucomicrobia, *Akkermansia*, *Bacteroides*, metabolites PC, PE, and serum hormone GH. Therefore, they can be further explored as candidate microbiota and metabolites for reducing tail fat deposition, in order to elucidate the mechanism of action, and provide new technical insights for reducing fat weight in the tail of large-tailed sheep, so as to improve its economic value.

## Data Availability

Data are available in a publicly accessible repository. The data presented in this study are openly available at NCBI, PRJNA826719.
